# Interactions Between Plant-Derived Psychoactive Substances and *Escherichia coli*

**DOI:** 10.3390/molecules31050893

**Published:** 2026-03-08

**Authors:** Joanna Wróblewska, Anna Długosz, Martyna Modrzejewska, Marcin Wróblewski, Damian Czarnecki, Alina Woźniak

**Affiliations:** 1 Department of Medical Biology and Biochemistry, Faculty of Medicine, Ludwik Rydygier Collegium Medicum in Bydgoszcz, Nicolaus Copernicus University in Toruń, 24 Karłowicza St., 85-092 Bydgoszcz, Poland; joanna.wroblewska@cm.umk.pl; 2 Department of Food Industry Technology and Engineering, Faculty of Chemical Technology and Engineering, Bydgoszcz University of Science and Technology, 3 Seminaryjna St., 85-326 Bydgoszcz, Poland; anna.dlugosz@pbs.edu.pl; 3 Department of Geriatrics, Division of Biochemistry and Biogerontology, Faculty of Health Sciences, Collegium Medicum in Bydgoszcz, Nicolaus Copernicus University in Toruń, 3 Dębowa St., 85-626 Bydgoszcz, Poland; martyna.modrzejewska@cm.umk.pl; 4 Department of Preventive Nursing, Faculty of Health Sciences, Ludwik Rydygier Collegium Medicum in Bydgoszcz, Nicolaus Copernicus University in Toruń, 1 Łukasiewicza St., 85-821 Bydgoszcz, Poland; czarneckidamian@cm.umk.pl

**Keywords:** cannabinoids, cocaine, *Escherichia coli*, opioids, psychoactive substances

## Abstract

Naturally occurring psychoactive substances, such as opioids, cocaine, and cannabinoids, affect not only the central nervous system but also the functioning of the microbiota–gut–brain axis. Available evidence indicates that their use is associated with changes in the gut microbiota and modulation of immune responses. *Escherichia coli*, a permanent component of the gut microbiota under conditions favoring dysbiosis, can enhance inflammatory responses and influence neuroimmunological mechanisms related to the development of addiction. This study aims to review and analyze the available literature concerning the effects of selected naturally derived psychoactive substances on *E. coli* and on the functioning of the microbiota–gut–brain axis, with particular emphasis on inflammatory processes and their potential significance in the pathogenesis of addiction.

## 1. Introduction

Naturally derived psychoactive substances exhibit remarkable chemical diversity and may originate from three primary sources: plants, microorganisms, and animals. A large number of plant species are known to possess confirmed or putative psychoactive properties [1]. The most widely recognized natural psychoactive compounds that have been or continue to be used in medicine include cocaine (historically), opioids, and cannabinoids.

Cocaine belongs to the group of psychostimulants, that is, substances that exert stimulatory effects on the central nervous system (CNS), and its use is associated with a high risk of abuse and the development of dependence. The mechanism of its pharmacological activity involves inhibition of dopamine reuptake and of other neurotransmitters, such as norepinephrine and serotonin (5-hydroxytryptamine, 5-HT), resulting in increased synaptic concentrations of these neurotransmitters and enhanced signal transmission between neurons [2,3,4]. Cocaine, as a plant-derived alkaloid, is obtained from coca leaves belonging to two cultivated species: *Erythroxylum coca* Lam. and *Erythroxylum novogranatense* (D. Morris) Hieron. These species arose through independent domestication processes and do not occur in the wild in a natural form [5]. As a potent stimulant, cocaine most commonly occurs as the hydrochloride salt intended for intranasal administration or in the form of a free base (so-called crack), used via inhalation [6].

Opioids constitute another critical group of psychoactive substances of significant clinical relevance. Chemically, they are structurally similar to natural alkaloids present in opium obtained from the opium poppy (*Papaver somniferum* L.) [2]. A classic example of a natural opioid is morphine, the principal alkaloid of the opium poppy, which exhibits vigorous analgesic activity and remains one of the cornerstone drugs used in the treatment of severe pain [7]. It belongs to the group of narcotic–sedative agents [1].

Industrial hemp and Indian hemp represent distinct chemotypes of *Cannabis sativa* L., producing more than 400 secondary metabolites, over 60 of which exhibit cannabinoid activity. Cannabinoids constitute a group of chemical compounds widely used worldwide in both cultural and therapeutic contexts. It should be emphasized that the two *Cannabis* chemotypes differ in their chemical profiles, including the content of the psychoactive compound tetrahydrocannabinol (THC) [2,8]. Other bioactive substances present in these plants include cannabidiol (CBD), cannabichromene, cannabigerol, cannabinol, and cannabidivarin [9]. According to the legal definition in force in the United States, the term “marijuana/hemp” encompasses all parts of these plants, their seeds, resin, and derivatives, excluding mature stalks, fibers, and non-viable seeds [10]. In the literature, *C. sativa* is described as a substance with sedative properties and mild hallucinogenic effects, the nature of which depends on the dose and the cannabinoid profile [1]. Products derived from these plants are used to treat numerous disease entities, which will be discussed in subsequent sections of this work. However, the presence of psychoactive compounds also determines their effects on the CNS, necessitating consideration of both their therapeutic potential and the risk of abuse. An increasing body of evidence indicates that the abuse of psychoactive substances is associated with the functioning of the microbiota–gut–brain axis and with the occurrence of comorbid conditions such as stress, anxiety, and depression, which are recognized as significant risk factors for the development of addiction [11]. Particular attention has also been directed toward the phenomenon of opioid-induced dysbiosis, which is associated with the development of numerous disorders and increasing tolerance to opioids [12]. One of the significant consequences of long-term opioid exposure is impaired gastrointestinal function, including compromised intestinal barrier integrity, bacterial translocation, and alterations in the profile of metabolites produced by the gut microbiota [2]. Increasing attention is also being paid to the potential impact of phytocannabinoids on the functioning of Gram-negative bacteria, including their involvement in bacterial stress responses and their possible role in modulating bacterial susceptibility to antibiotics [13,14].

The gut microbiota plays a key role in maintaining organismal homeostasis and in the pathogenesis of many diseases. It exerts its effects by regulating epigenetic, metabolomic, and immunological processes, as well as by modulating CNS function, through dynamic, bidirectional communication within the so-called microbiota–gut–brain axis. Microorganisms colonizing the gastrointestinal tract are responsible for maintaining local environmental homeostasis, carrying out key metabolic processes, and supporting immune defense mechanisms [12,15]. The human colon is inhabited by an enormous number of microorganisms, with bacterial densities reaching 10^11^–10^14^ cells per gram of intestinal content, forming a complex ecosystem comprising over 1000 bacterial species [16,17].

Bacteria belonging to the family *Enterobacteriaceae* constitute a small fraction of the normal human gut microbiota, typically accounting for less than 1% of the total bacterial population. Despite their relatively low abundance, these bacteria perform critical physiological functions whose significance has not yet been fully characterized [16]. Of particular importance is *Escherichia coli*, which, as a facultative anaerobe, plays a significant ecological role in the gut, including the consumption of residual oxygen and thereby creating conditions favorable for the growth of obligate anaerobes, as well as participating in the synthesis of selected bioactive compounds, including vitamin K, biotin [16]. Although *E. coli* is a permanent component of the gut microbiota, certain intestinal strains equipped with specific virulence factors may, under favorable conditions, breach the intestinal barrier, undergo translocation to physiologically sterile sites, and induce an inflammatory response [18]. It should be noted that bacterial translocation also occurs in the case of intestinal barrier damage [19]. As a result, both the presence of specific virulence factors in *E. coli* and impairment of the intestinal barrier may promote the development of gastrointestinal and extraintestinal infections of endogenous origin induced by this bacterium. Additionally, it should be emphasized that the early period of human development is critical for the maturation of the nervous and immune systems. Infections experienced during this time may lead to long-term alterations in the way the organism responds to various challenges encountered later in life. The scientific literature increasingly emphasizes that early immune activation can permanently alter brain function, affecting both inflammatory processes and neuronal and glial cell activity [20].

The present study aims to review and analyze the available literature data concerning the effects of selected naturally derived psychoactive substances on *E. coli* and on the functioning of the microbiota–gut–brain axis, with particular emphasis on the role of this bacterium in modulating inflammatory responses, intestinal barrier integrity, and neuroimmunological mechanisms associated with the development of addiction. Furthermore, this work aims to present selected biological characteristics of *E. coli* relevant to its role in the gut microbiota and its interactions with the host under conditions of exposure to psychoactive substances.

## 2. Therapeutic and Non-Therapeutic Uses of Selected Psychoactive Substances

The use of legal opioids can be therapeutic and non-therapeutic. Non-therapeutic use of opioids often involves morphine, fentanyl, loperamide, or codeine syrups. However, the use of illegal opioids (non-therapeutic) usually concerns heroin.

Opioids are primarily known for their analgesic and sedative effects and remain one of the main classes of drugs used in the treatment of acute, postoperative, and cancer-related pain. At the same time, their use is associated with a high risk of misuse, the development of tolerance, and addiction. The mechanism of opioid action is based on their binding to specific opioid receptors (μ, κ, and δ), which belong to the family of G protein–coupled receptors and are located on the surface of neurons as well as non-neuronal cells [2]. These receptors are expressed not only in the central nervous system, where they mediate analgesia, euphoria, and respiratory depression, but also in the peripheral nervous system, the gastrointestinal tract, and the ENS [12].

The presence of opioid receptors in the intestines is of significant clinical importance, as their activation leads to inhibition of peristalsis, reduced intestinal secretion, and increased sphincter tone, thereby promoting the development of constipation and other gastrointestinal motility disorders [21,22]. However, increasing evidence indicates that the effects of opioids on the gastrointestinal tract extend beyond motor functions and also include modulation of intestinal epithelial barrier integrity, immune system function, and the composition of the gut microbiota [12,23]. These mechanisms may indirectly contribute to the development of chronic inflammation, increased intestinal permeability, and bacterial translocation, which are particularly relevant in the context of inflammatory bowel diseases (IBD) and systemic infectious complications [24,25].

Opioid drugs are commonly used in addiction treatment. For example, buprenorphine and methadone are used in the substitution therapies [26]. Buprenorphine and methadone are used as medications that, in addition to therapeutic effects, also produce side effects, including those affecting the digestive system. Buprenorphine causes abdominal pain, diarrhea, indigestion, and dry mouth [27,28]. Moreover, buprenorphine can be used to treat disorders related to loperamide use, and its effectiveness is most significant when patients are in mild to moderate withdrawal stages [29].

Cocaine, despite its historical use in medicine as a local anesthetic, is no longer used routinely for therapeutic purposes due to its high addictive potential and systemic toxicity [4,6]. Its relevance in the context of the present work arises primarily from its non-therapeutic use and its well-documented effects on the CNS and immune responses, which may, in turn, indirectly influence the functioning of the microbiota–gut–brain axis [30]. Statistical data show that crack use is increasing in Europe, particularly among socially marginalized groups. Treatment for cocaine use disorders has limited effectiveness. There are attempts to use cocaine-assisted treatment, similar to heroin-assisted therapy. These are harm reduction methods [31]. This means that cocaine addiction is a major social problem, and attempts are being made to introduce addiction treatment methods as a form of harm reduction involving cocaine use. Some parts of society in many countries are exposed to the effects of cocaine.

Cannabinoids represent another critical group of psychoactive substances with growing therapeutic relevance [1]. Their biological effects are primarily mediated by the cannabinoid receptor type 1 (CB1) and cannabinoid receptor type 2 (CB2) [32]. The CB1 receptor is predominantly expressed in the central nervous system. It is responsible for the psychoactive effects of THC. In contrast, the CB2 receptor is expressed mainly in immune system cells and glial cells, including microglia and astrocytes, and to a lesser extent also in neurons [33,34]. Compounds that interact with the CB2 receptor have attracted particular interest as potential therapeutic and/or preventive agents for the treatment of neuropathic pain, neuroinflammation, and neurodegenerative diseases [35,36].

In the central nervous system, modulation of CB2 activity exhibits pronounced neuroprotective effects. Preclinical studies indicate that CB2 activation may produce beneficial outcomes in the course of neurodegenerative diseases, including Alzheimer’s disease, where reductions in tau protein phosphorylation, protection against the neurotoxic effects of β-amyloid, and attenuation of microglial activation have been observed. Similar neuroprotective effects have been reported in models of Parkinson’s disease, including reduced neurodegeneration and neuroinflammation, as well as in Huntington’s disease, where protection of striatal neurons and suppression of inflammatory responses within the CNS have been demonstrated. Moreover, available reports suggest that cannabis and cannabinoids may contribute to the alleviation of motor symptoms and to the reduction in behavioral and psychological symptoms of dementia [37]. An increasing body of evidence also points to a significant role of the endocannabinoid system in the regulation of gastrointestinal function, including control of intestinal motility, epithelial barrier integrity, and mucosal immune responses, which may be relevant in the course of IBD and disorders of the microbiota–gut–brain axis [38]. Endocannabinoids cause the release of neurotransmitters from enteric neurons and regulate peristaltic contractions and secretory responses, which are crucial for efficient digestion [38]. In this context, attention has been drawn to the ability of phytocannabinoids to modulate host–microbe interactions, including their potential antimicrobial activity and effects on colonization by antibiotic-resistant bacteria [13,14]. CBD is the most extensively studied naturally occurring cannabinoid and the dominant cannabinoid in industrial hemp. It is attributed with anticonvulsant, analgesic, anxiolytic, neuroprotective, antioxidant, and antimicrobial properties. The Food and Drug Administration has approved Epidiolex^®^, GW Pharmaceuticals Limited, Cambridge, UK (CBD isolate as the active substance) for the treatment of epileptic seizures associated with Lennox–Gastaut syndrome and Dravet syndrome in pediatric patients [9,39]. The preparation Sativex^®^, GW Pharmaceuticals Limited, Cambridge, UK (containing a combination of CBD and THC) may be used in the treatment of moderate to severe spasticity associated with multiple sclerosis [39]. Cannabinoid-containing products show potential applications in the treatment of various dermatological conditions, such as acne vulgaris, allergic contact dermatitis, pruritus, psoriasis, and skin cancers; however, available data on their safety and efficacy remain limited [9]. Research indicates that CBD plays a role as an adjunct therapy in the treatment of opioid use disorder, during both opioid use and withdrawal. Studies on humans have shown a reduction in cravings and minimization of anxiety during withdrawal syndrome. CBD has been shown to reduce withdrawal symptoms and minimize the rewarding effects of opioids [40]. Hurd et al. [41] conducted a clinical study to examine the therapeutic effects of CBD in people with opioid use disorder. Participants received 400 or 800 mg of CBD per day for 3 days. It was shown that CBD reduced opioid cravings and anxiety in the study participants, suggesting a new method for treating opioid use disorder [40].

## 3. The Effects of Selected Psychoactive Substances on the CNS: From Functional Changes to Mechanisms of Addiction

Endogenous opioid pathways, endogenous cannabinoids, and orexin signaling are involved in the hedonic reward of food consumption [42]. Hedonic hunger may influence eating behaviors. Eating behaviors are influenced by taste preferences, as well as pleasurable experiences in the brain’s reward system, which are triggered by the anticipation of consuming tasty foods. This can trigger the release of dopamine in brain areas associated with reward and evoke thoughts and cravings for food [42]. Gut microbiota can produce proteins called peptidomimetics. These proteins mimic those that regulate hunger, such as peptide YY, leptin, and ghrelin [42]. One such protein is caseinolytic peptidase B (ClpB), produced by *E. coli*, which acts similarly to the hunger-suppressing hormone α-melanocyte-stimulating hormone (α-MSH). Animal studies have shown that, like α-MSH, ClpB increases levels of peptide YY or GLP-1 in the body and activates hypothalamic neurons that inhibit hunger. This indicates that gut microbiota can suppress hunger through peptidomimetics [42]. The brain’s reward system plays a key role in the process of addiction to psychoactive substances. This may suggest that natural mechanisms regulating hunger, including the microbiome, can influence drug craving, and that the intake of certain psychoactive substances can modulate these mechanisms (positively or negatively).

The use of psychoactive substances is associated with a risk of developing addiction, which constitutes a complex disorder with a neurobiological basis, involving persistent functional and structural changes within the central nervous system. A key role in the pathogenesis of addiction is attributed to the mesocorticolimbic system, which includes the ventral tegmental area (VTA), the nucleus accumbens (NAc), the amygdala, the hippocampus, and the prefrontal cortex. This system integrates signals related to reward, motivation, associative learning, and stress [43]. Prolonged exposure to psychoactive substances leads to neuroadaptations within these structures, resulting in the development of tolerance, withdrawal symptoms, and compulsive substance seeking despite negative consequences [43,44,45].

In the context of addiction, activation of μ-opioid receptors (MOR) within the mesocorticolimbic reward system plays a key role. By inhibiting gamma-aminobutyric acid (GABA) ergic interneurons in the ventral tegmental area (VTA), opioids disinhibit dopaminergic neurons and increase dopamine release in the nucleus accumbens, resulting in strong positive reinforcement [2,44]. Chronic exposure to opioids, however, induces numerous neuroadaptations, including changes in the expression and sensitivity of opioid receptors, disturbances in glutamatergic neurotransmission, and modifications of synaptic plasticity within the prefrontal cortex and limbic structures. These processes underlie the development of tolerance, withdrawal symptoms, and loss of control over substance intake [43]. Increasing evidence also indicates a significant role of neuroinflammation and microglial activation in opioid addiction, which may contribute to the severity of affective symptoms, increased stress vulnerability, and a higher risk of relapse [46].

Cocaine belongs to the group of potent psychostimulants whose effects are primarily mediated by inhibition of monoamine reuptake, particularly dopamine, through blockade of the dopamine transporter [2]. This results in a rapid increase in dopamine concentration within the synaptic cleft of the reward system and the induction of intense euphoric effects. This mechanism promotes the rapid development of addiction and strong behavioral reinforcement [43].

During long-term cocaine use, persistent changes are observed in the functioning of the prefrontal cortex, which is responsible for cognitive control and impulse inhibition, as well as in the nucleus accumbens and the amygdala. These alterations facilitate the consolidation of responses to cues associated with substance use and increase the risk of relapse [43]. Contemporary studies also indicate the involvement of neuroinflammatory processes and changes in the expression of genes related to synaptic plasticity in maintaining pathological patterns of cocaine-related behaviors [47,48,49].

The effects of cannabinoids on the CNS are primarily mediated by CB1 receptors, which are widely expressed in brain structures involved in the regulation of mood, motivation, memory, and reward perception [50]. Activation of CB1 receptors by THC is responsible for the psychoactive effects of cannabis, as well as for the development of tolerance and withdrawal symptoms observed in some users [50]. Chronic THC use leads to desensitization and downregulation of CB1 receptors and to disturbances in the balance between GABAergic and glutamatergic neurotransmission, which may affect the functioning of the reward system and promote the development of cannabis use disorder [51,52]. Withdrawal symptoms, such as irritability, anxiety, sleep disturbances, and reduced appetite, may further reinforce repeated substance use through mechanisms of negative reinforcement [43]. At the same time, increasing interest has been directed toward the role of CB2 receptors in the central nervous system, particularly in the context of neuroinflammation and neurodegeneration. Activation of CB2 receptors, which are primarily expressed in microglia and astrocytes, may modulate inflammatory responses, exert neuroprotective effects, and limit the psychoactive effects characteristic of CB1 receptor activation [50]. This phenomenon is of considerable importance in the search for therapeutic strategies aimed at minimizing the risk of addiction.

Despite differences in the primary molecular targets of opioids, cocaine, and cannabinoids, their long-term use leads to partially shared neurobiological consequences, including dysfunction of the reward system, impaired executive control, and an increased contribution of stress and inflammatory processes within the CNS [43]. Growing evidence also suggests that these mechanisms may be modulated by immune signaling and interactions within the microbiota–gut–brain axis, providing an essential context for the subsequent sections of this work devoted to the gut microbiota and the role of *E. coli* [43,53].

## 4. Structure of the Intestinal Barrier and Enteric Nervous System

Enteroendocrine cells (EECs) are primarily found in the intestinal epithelium of the small intestine. EECs regulate gastrointestinal function and homeostasis by the secretion of many peptides and hormones, such as glucagon-like peptide-1, peptide YY, cholecystokinin, ghrelin, melatonin, neurotensin, and serotonin in response to mechanical, nutritional, and microbial signaling [54,55,56,57]. EECs mediate communication between the gut microbiota and the brain via endocrine signals, neural pathways, and the gut immune system in the gut–brain axis [58,59,60].

**Figure 1:**
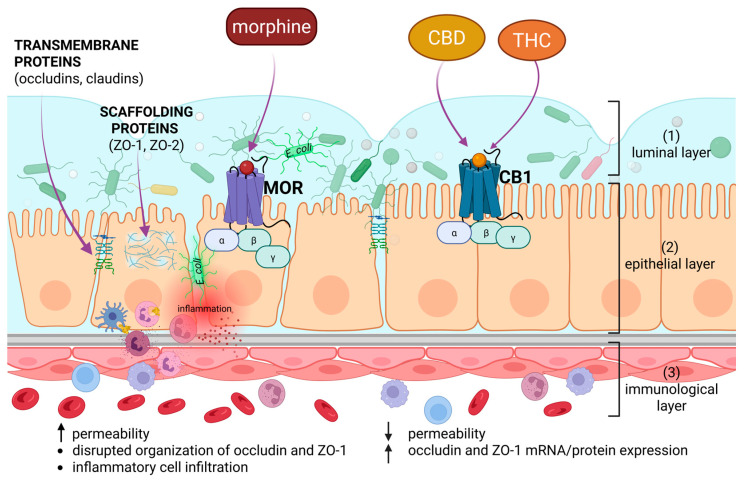
Simplified illustration of the different effects of plant-derived psychoactive substances (cannabinoids and opioids) on the intestinal barrier integrity. CBD, cannabidiol; THC, tetrahydrocannabinol; CB1, type 1 of cannabinoid receptor; MOR, µ-opioid receptor; ZO-1, ZO-2, zona occludens-1,2 proteins. The black up arrow indicates an increase. The black down arrow indicates a decrease. The purple arrow indicates the place of action of a specific compound or the occurrence of a protein. *E. coli* is marked in light green. The number in parentheses indicates the individual layer of the intestinal barrier, described in more detail in the text. Created in BioRender. Modrzejewska, M. (24 February 2026) https://BioRender.com/q4snzzp.

The small intestinal barrier has three layers: (1) the luminal layer consisting of non-pathogenic bacteria and mucus, (2) the epithelial monolayer built of enterocytes reinforced by cell junctions, and (3) the immunological layer constituted by immune cells in the lamina propria (Figure 1) [61]. The epithelial physical barrier, supported by the secretion of antimicrobial peptides (such as lysozyme and defensins), prevents the invasion of the human body by pathogenic organisms and/or the detrimental overgrowth of commensal bacteria [62]. Its permeability is regulated by i.a. the tight junction transmembrane proteins (such as claudins, occludins), which seal paracellular space between enterocytes, and zona occludens-1,2 (ZO-1, ZO-2), which constitute the scaffolding molecules [23,63]. The integrity of the intestinal barrier maintains gut and whole-body health. In contrast, its disruption can lead to conditions such as irritable bowel syndrome (IBS), IBD, or pseudomembranous colitis [57,64]. IBD or IBS may also be caused by disturbances in the ENS [65,66]. It is composed of the Meissner’s and Auerbach’s nerve plexuses, which run from the human’s esophagus to the anus. The ENS is called our “second brain,” and it acts independently of the CNS. The ENS communicates with the CNS through the sympathetic and parasympathetic parts of the autonomic nervous system. The ENS consists of non-neuronal cells (enteroglial cells, EGCs) [67], interneurons, and motor and sensory neurons [68,69,70]. EGCs (astroglial and microglial cells) function similarly to brain glial cells, helping maintain ENS homeostasis. Moreover, they can proliferate and activate in response to gut injury and inflammation, leading to enterogliosis [71]. EGCs communicate with other infiltrating immune cells, such as neutrophils, macrophages, and mast cells, by releasing growth factors (neurotrophins) and cytokines [72,73].

Stimuli from the digestive tract, such as the presence of chyme or gut distension, are detected by sensory neurons, then processed by interneurons, and transmitted to motor neurons, which elicit the final response [57]. The ENS coordinates intestinal processes such as motility and peristalsis, nutrient detection, the immunological response, intestinal barrier function, and the vasculature of the gastrointestinal tract, as well as epithelial and glandular secretion [68]. The neurons of the ENS are subject to stimulation by acetylcholine (ACh) and substance P, as well as inhibition by dopamine, nitric oxide, vasoactive intestinal peptide, GABA, ATP, and adenosine. Serotonin and calcitonin gene-related peptide (CGRP) act as sensory neurotransmitters [57].

### The Influence of Selected Psychoactive Substances on the Integrity of the Intestinal Barrier

Endocannabinoids (anandamide, AEA, and 2-arachidonylglycerol, 2-AG) impact peristalsis via CB1 and CB2 receptors, as well as the transient receptor potential vanilloid-1 (TRPV1). Cannabinoid receptors are G protein-coupled receptors located presynaptically. They exert influence by modulating synaptic neurotransmission of ACh, glutamate, and GABA [74,75]. Plant-derived cannabinoids such as CBD and THC affect intestinal motility mainly through the CB1 receptor [76,77], while CBD has limited affinity for CB1/2 receptors [57]. Most cannabinoids act on the TRPV1 receptor in a two-phase manner. First, they activate the receptor, which then leads to its desensitization. Both AEA and CBD are TRPV1 agonists [78]. In addition, CBD exerts its anti-inflammatory and antioxidant effects through 5-HT 1A receptors and peroxisome proliferator-activated receptor gamma [79,80]. It has been demonstrated that CB1 receptors are abundant in the human brain [73], while in the gastrointestinal tract their distribution focuses on neurons of the ENS (motor neurons, interneurons, and intrinsic primary afferent neurons), epithelial cells, and sensory endings of vagal and spinal neurons. Activation of the CB1 receptor is associated with a reduction in gastric acid secretion, gastric emptying, and intestinal motility [74].

Transepithelial electrical resistance (TEER) is a non-invasive and quantitative method for assessing the integrity of cellular monolayers. Phytocannabinoids, including CBD and THC, applied to the apical or basolateral membrane of Caco-2 cells decreased permeability in the EDTA-treated layer. On the other hand, the influence of endocannabinoids depended on epithelial cell polarization. The application of AEA and 2-AG to the apical compartment increased permeability, whereas their application to the basolateral membrane restored the integrity of the EDTA-treated layer, accompanied by TRPV1 activation. The authors showed that all tested cannabinoids increased ZO-1 mRNA levels, suggesting that they attenuate EDTA-induced intestinal barrier destruction. Simultaneously, endocannabinoids also reduced claudin-1 mRNA levels, underscoring their role in modulating intestinal permeability [77].

Enterotoxin A inactivates small GTPases, i.e., RhoA, which is a crucial regulator of cytoskeletal structure and tight junction function. Inactivation of RhoA results in a transition from the GTP-bound to GDP-bound form, affects cellular structure, tight junction integrity, and, consequently, increases the epithelial barrier permeability. This cascade of molecular events intensified by colitis can lead to leaky gut [64,81]. Exposure to toxin A caused a significant, time-dependent reduction in TEER in Caco-2 cell layers. In turn, the destructive influence of *C. difficile* toxin A on cultured cells was significantly counteracted, depending on CBD concentration. It was shown that, in enterotoxin A-treated Caco-2 cells, reduced ZO-1 protein expression was significantly restored by CBD in a concentration-dependent manner. These results were also confirmed by immunofluorescent staining [64].

Histological analysis of small intestine and colon tissue samples from 24 h morphine-treated mice revealed a harmful effect of this opioid, expressed by inflammatory cell infiltration. Morphine disrupted the organization of occludin and ZO-1, affecting only small intestinal villi but not the colon. The authors also showed that morphine increases intestinal permeability (as determined by whole-blood FITC-dextran concentration) in a MOR-dependent manner. This opioid alters the distribution of tight junction proteins rather than their expression levels. Significantly, the above-mentioned changes did not affect colonic epithelium, suggesting a distinct mechanism for regulating its barrier function [23]. The possible influence of the plant-derived psychoactive compounds on the integrity of the intestinal barrier described in this subchapter is presented in Figure 1.

The TRPV1 receptor is a non-selective cation channel that can be activated in response to capsaicin, pro-inflammatory mediators, acidic environment, and elevated temperature during inflammation or infection [82]. Activation of TRPV1 receptors located on primary sensory afferent neurons in the intestine is followed by intracellular influx of calcium and sodium ions. This causes depolarization, neuronal hyperexcitability, and pain sensation. Activated nerves release substance P and CGRP, which are responsible for vasodilation, attracting immune cells, and causing swelling, leading to the so-called neurogenic inflammation [83,84]. The TRPV1 receptor plays a significant role in the pathophysiology of IBS, contributing to the visceral hypersensitivity (pain during normal bowel function, e.g., stretching due to gas), motility disorders (diarrhea or constipation), and abdominal pain. In colonic biopsies from 23 IBS patients, TRPV1-expressing nerve fibres were 3.5-fold more frequent than in 22 controls (*p* < 0.0001) [85].

**Figure 2:**
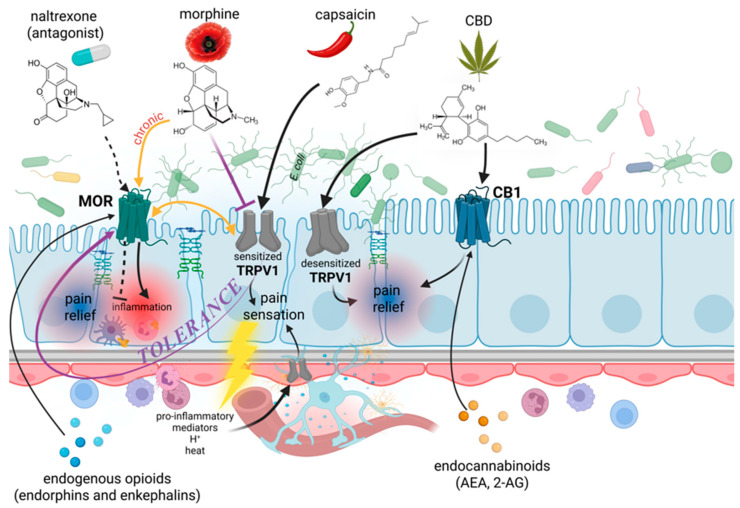
The interplay between opioid and cannabinoid pathways, with particular emphasis on the interactions between MOR, CB1, and TRPV1 receptors in the bowel. The figure also presents the possible influence of selected psychoactive substances of plant or endogenous origin on visceral hypersensitivity and inflammation, which may facilitate the invasion of intestinal bacteria, including *E. coli*. 2-AG, 2-arachidonylglycerol; AEA, anandamide; CB1, type 1 of cannabinoid receptor; CBD, cannabidiol; H^+^, hydrogen ions; MOR, µ-opioid receptor; TRPV1, transient receptor potential vanilloid-1. Created in BioRender. Modrzejewska, M. (26 February 2026) https://BioRender.com/kg2lm8r.

It has been suggested that the cannabinoid and opioid pathways, particularly the CB1 and MOR receptors, are physiologically linked to TRPV1 in sensory fibers [84,86], as schematically presented in Figure 2. Protein kinase-dependent (e.g., cAMP-dependent protein kinase A, PKA, or protein kinase C, PKC) phosphorylation causes receptor sensitization, while dephosphorylation causes its desensitization. The phosphorylation status of TRPV1 may depend on specific pathological conditions. For example, morphine inhibits PKA phosphorylation, which blocks TRPV1 receptor sensitization. Although an indirect interaction of TRPV1 receptors with morphine may initially help with pain relief, it was reported that MOR-mediated decrease in cAMP level (adenylyl cyclase inhibition) affects PKA activity and limits the action of TRPV1 [86,87], while chronic MOR stimulation leads to TRPV1 hyperactivity. This, in turn, weakens the analgesic effects of morphine, forcing the use of increasingly higher doses and resulting in the development of tolerance (Figure 2). MOR activation triggers intracellular signaling pathways (via PKC or PKA) that phosphorylate the TRPV1 channel. Phosphorylated TRPV1 opens more readily (at lower stimulus concentrations), sending pain signals to the brain despite morphine administration. It is related to the activation of Ca^2+^/calmodulin-dependent protein kinase II and the cAMP response element binding protein (CREB) pathway in the nucleus accumbens, which constitute the foundation of behavioral and physical addiction [88]. In contrast, the synthetic cannabinoid WIN55, via calcineurin, promotes dephosphorylation and hence receptor desensitization [89]. Moreover, some studies suggest that CBD modulation of TRPV1 may improve the integrity of the gut barrier, preventing toxins and bacteria from entering the bloodstream [90,91].

Endogenous opioid peptides (endorphins and enkephalins) are believed to be involved in the development of inflammation [92,93]. The above-mentioned μ, κ, and δ-opioid receptors may also be expressed on immune cells [94,95]. Smith et al. [96] hypothesized that the endogenous opioid system is involved in IBD, and the use of the opioid receptor antagonist, naltrexone, may reduce the inflammatory process (Figure 2). They clearly demonstrated that naltrexone administered orally for 12 weeks caused mucosal healing, as confirmed by histological examination in 18 patients with moderate to severe Crohn’s disease compared with 16 patients receiving placebo (*p* = 0.008).

## 5. Selected Aspects of the Biology of *E. coli* in Interactions with the Host Organism and Environmental Factors

*E. coli* exhibits characteristics of facultative pathogens, meaning that under favorable conditions, it can both colonize the gastrointestinal tract as a commensal and cause disease [97]. Its metabolic adaptations enable efficient utilization of available resources within the intestinal niche, located in the mucus layer of the cecum and colon, thereby promoting a competitive advantage over other intestinal microorganisms. Pathogenic *E. coli* strains are classified into intestinal pathogenic *E. coli* and extraintestinal pathogenic *E. coli* (ExPEC). Both groups comprise distinct pathotypes, understood as sets of strains within a single species characterized by specific pathogenic properties [98]. ExPEC strains harbor a set of virulence factors that facilitate bacterial colonization of the host, followed by invasion and dissemination. These determinants are encoded both within the bacterial chromosome, most often in structures referred to as pathogenicity islands, and on plasmids. The most important virulence factors include adhesion factors, iron acquisition systems, mechanisms that enable evasion of the host immune response, and toxins [99]. The interaction between these virulence factors and host tissues leads to the activation of innate immune mechanisms that recognize bacterial components and initiate the inflammatory response. Toll-like receptors (TLRs) are transmembrane proteins that sense pathogen-associated molecular patterns such as lipoproteins, lipopolysaccharides (LPS), DNA, and ssRNA. TLRs induce the secretion of inflammatory cytokines in innate immune myeloid cells, stimulating lymphocytes to mount an antigen-specific adaptive immune response that ultimately clears invading pathogens [100]. TLRs, as a fundamental part of innate immunity, together with the intestinal epithelium, provide a balanced response to stimulation by the commensal microbiota in the intestinal lumen. The attachment of bacterial products to TLRs on enterocytes promotes enterocyte proliferation, the expression of antimicrobial peptides, and IgA secretion into the gut lumen [101]. A complex cell envelope structure characterizes Gram-negative bacteria. The cytoplasmic membrane has a classical phospholipid bilayer structure. In contrast, the outer membrane exhibits an asymmetric organization, in which the inner leaflet is composed of phospholipids and the outer leaflet of LPS. LPS functions as an endotoxin and consists of three main components: lipid A, which is responsible for endotoxin activity; an oligosaccharide core; and the O antigen, which determines the serological variability of bacteria and plays a vital role in interactions with the host immune system [102]. It is worth emphasizing that the presence of an outer membrane containing LPS constitutes a significant barrier to many hydrophobic compounds. Given the lipophilic nature of CBD, available data confirm that its antibacterial activity against Gram-negative bacteria, including *E. coli*, is limited [103,104]. LPS is recognized by pattern recognition receptors, among which Toll-like receptor 4 (TLR4) plays a key role. LPS recognition involves lipopolysaccharide-binding protein, which transfers the endotoxin to the CD14 receptor and subsequently to the Toll-like receptor 4–myeloid differentiation factor 2 complex on the surface of antigen-presenting cells, such as macrophages. Activation of this complex initiates a signaling cascade leading to the activation of the transcription factor nuclear factor kappa B (NF-κB) and the induction of genes encoding pro-inflammatory cytokines, which constitutes a key element of the innate immune response to Gram-negative bacterial infections [105,106]. In healthy individuals, the inflammatory response to LPS is characterized by an initial intense production of pro-inflammatory cytokines, followed by the development of mechanisms that limit further inflammatory activation. This phenomenon is referred to as TLR signaling reprogramming or endotoxin (LPS) tolerance. The mechanisms underlying endotoxin tolerance include, among others, silencing of key mediators of TLR signaling, impaired interactions among signaling cascade components, and overexpression of negative regulators such as A20 [107].

Between the cytoplasmic membrane and the outer membrane lies the periplasmic space, which contains a thin layer of peptidoglycan as well as numerous periplasmic proteins that perform essential structural and metabolic functions in Gram-negative bacterial cells. The structural and functional linkage between the outer membrane and the periplasmic space enables the formation of outer membrane vesicles (OMVs), which are spherical structures released by Gram-negative bacteria during growth [102]. OMVs play an essential role in bacterial communication, and their production is associated with bacterial responses to stress conditions. In recent years, increasing attention has been paid to their potential involvement in the development of bacterial antibiotic resistance mechanisms. Studies by Kosgodage et al. [13] demonstrated that CBD significantly inhibits the release of OMVs by *E. coli* strains. In addition, it was shown that CBD can modulate (increase) the susceptibility of *E. coli* to antibiotics not routinely used in the treatment of these infections, such as erythromycin, rifampicin, and vancomycin.

Antibiotic resistance is not solely associated with molecular mechanisms occurring within bacterial cells but is closely linked to environmental factors and human behavior. Barani et al. [14] demonstrated a high prevalence of intestinal carriage of *E. coli* strains producing extended-spectrum β-lactamases (ESBL) among individuals addicted to drugs. The analyzed population included users of opium and its derivatives, amphetamine, and methamphetamine, with opioid users constituting the dominant group. In more than half of the isolates exhibiting the ESBL phenotype, resistance genes were detected, predominantly *bla-TEM*, as well as *bla-CTX-M* and *bla-SHV*. The authors emphasize that the intestinal microbiota of drug users, due to inappropriate use of medications and poorer hygienic conditions, may represent a significant source of the dissemination of resistance genes among both commensal and pathogenic bacteria, posing a serious public health threat.

## 6. Role of *E. coli* in the Modulation of the Gut–Brain Axis and Addiction Mechanisms

The gut microbiome plays a crucial role in maintaining gastrointestinal homeostasis in humans, among others, by producing bioactive metabolites and limiting intestinal colonization by pathogenic microorganisms. Disruption of this balance, referred to as dysbiosis, leads to a range of adverse changes, including reduced microbial diversity, a relative increase in potentially pathogenic bacteria, and a concomitant decrease in beneficial commensal bacteria [17]. Although studies in humans are essential for translational analyses of the gut–brain axis, the majority of mechanistic knowledge derives from animal models, which allow controlled, precise manipulation of the gut microbiome and appropriate behavioral testing. Findings from studies using germ-free animals and fecal microbiota transplantation indicate that the composition of the gut microbiome significantly modulates stress responses and anxiety- and depression-like behaviors, thereby confirming its key role in regulating gut–brain axis functioning [17]. Experimental oral administration of the human-derived inflammatory *E. coli* K1 strain in mice induced colitis, increased blood LPS levels, hippocampal inflammation, cognitive decline, and depression-like behaviors, suggesting that inflammatory gut bacteria can influence intestinal and brain function [108]. The gut microbiota plays a key role in modulating central neurotransmission and brain function by influencing major neurotransmitter systems and metabolic pathways [109,110,111]. Mesolimbic circuits have been proposed as potential targets through which microbiome-related interventions may influence behavioral responses to drugs of abuse, including alcohol and opioids [17]. The concept of microbial endocrinology emphasizes that microorganisms inhabiting the gastrointestinal tract can produce and recognize the same neuroendocrine hormones as the host, thereby enabling bidirectional communication within the gut–brain axis [112]. This bidirectional communication is further supported by the identification of the two-component sensor kinase quorum-sensing *E. coli* regulator C (QseC) in *E. coli*, which has been shown to directly sense host-derived epinephrine and norepinephrine, undergo autophosphorylation, and transmit the signal to its cognate response regulator [112,113]. Biochemical analyses have demonstrated that QseC undergoes autophosphorylation upon exposure to epinephrine or norepinephrine and subsequently transfers the phosphate group to its response regulator quorum-sensing *E. coli* regulator B (QseB), thereby modulating transcriptional programs in *E. coli* [113].

Some gut bacteria, including *E. coli*, can produce neuroactive compounds such as norepinephrine and modulate tryptophan metabolism, which indirectly affects serotonergic neurotransmission. In this way, *E. coli* may participate in gut–brain axis signaling and indirectly influence CNS function [109]. It has been demonstrated that members of the gut microbiota can synthesize neurochemicals such as GABA, dopamine, norepinephrine, serotonin, acetylcholine, and histamine using biochemical pathways identical to those found in vertebrate tissues [112]. In *E. coli*, GABA is synthesized from glutamate by the pyridoxal-5′-phosphate-dependent enzyme glutamate decarboxylase (GadB), which forms part of the glutamate-dependent acid resistance system [114]. Capitani [114] showed that GadB activity is strongly pH-dependent and that the enzyme undergoes conformational rearrangements that regulate access to its active site. GadB is a hexameric enzyme and, together with the antiporter GadC, constitutes the glutamate-dependent acid resistance system that mediates proton consumption in *E. coli.* Exposure to cocaine may indirectly affect the composition of the gut microbiota by increasing intestinal norepinephrine levels, thereby promoting colonization by certain commensal bacteria, including *E. coli* [115]. Norepinephrine can modulate interactions between commensal and virulent *E. coli* strains, including O157:H7, and the intestinal epithelium, thereby promoting the early stages of bacterial adhesion to host cells [116]. The QseC-dependent signaling pathway regulates flagellar gene expression and contributes to virulence gene regulation in enterohemorrhagic *E. coli*, linking host catecholamine signaling with bacterial behavioral responses. Deletion of *qseC* attenuates virulence in a rabbit infection model, supporting the functional relevance of this signaling pathway in host–pathogen interaction [113].

Alterations in the composition of the gut microbiota may lead to modifications in the host metabolic profile, particularly to a reduction in glycine availability in the intestine, blood, and cerebrospinal fluid, which is associated with an exacerbation of behavioral responses, such as enhanced cocaine-induced locomotor activity. It has also been demonstrated that interference with microbiota-dependent glycine metabolism, including through glycine supplementation or the use of bacterial strains incapable of glycine uptake, reverses the observed behavioral changes, thereby confirming the role of the gut microbiota in modulating interactions between the gastrointestinal tract and the CNS in response to psychoactive substances [115]. Overall, the available evidence indicates that microbial metabolic activity may contribute to behavioral sensitivity to drugs of abuse rather than merely reflect secondary alterations [17].

In vitro studies using human cells complement data derived from animal models and indicate that specific gut bacteria may directly modulate the expression of genes associated with addiction. It has been shown that stimulation of the human intestinal epithelial cell line HT-29 with pathogenic *E. coli* strains (the intracellular strain *E. coli* UM146 and the extracellular strain *E. coli* UM147), particularly the intracellular strain, leads to significant changes in the expression of genes belonging to signaling pathways associated with cocaine and amphetamine addiction, including genes involved in cognitive functions (*GNAS*, *PPP1R1B*), stress responses (*JUN*, *CREB1*), and anxiety (*BDNF*). Concurrently, the observed downregulation of the *FOSB* gene, which participates in the organism’s response to morphine, suggests a potential association between bacterially induced changes in gene expression and mechanisms underlying opioid response [117]. Such alterations are consistent with evidence suggesting that immune and microbiota-related signaling pathways may influence brain function and reward-related processes implicated in addiction [11]. One mechanism potentially linking the gut microbiota, including Gram-negative bacteria such as *E. coli*, to the gut–brain axis and the pathophysiology of affective disorders and addictions is the activation of Toll-like receptors, particularly TLR4. Genetic studies in mice demonstrated that mutations in the *Lps* locus, identified as the *Tlr4* gene, abolish cellular responsiveness to LPS, indicating that TLR4 is essential for LPS signal transduction. In C3H/HeJ mice, a missense mutation in *Tlr4* results in substitution of proline with histidine at position 712 within the cytoplasmic domain of the receptor, whereas C57BL/10ScCr mice carry a null mutation associated with the absence of *Tlr4* mRNA expression. These findings established that TLR4 is required for recognition of LPS and for downstream signaling in response to Gram-negative bacteria [118]. Activation of TLR4 in the CNS may lead to microglial activation and increased production of proinflammatory cytokines, thereby promoting the development of neuroinflammation, which has been associated in numerous studies with the occurrence of depressive and anxiety-related symptoms [119]. Consistent with this pathway, oral administration of the inflammatory *E. coli* K1 strain in mice has been shown to increase fecal and circulating LPS levels, activate NF-κB signaling in the colon and hippocampus, elevate tumor necrosis factor alpha (TNF-α) and interleukin-6 (IL-6) expression, and increase ionized calcium-binding adaptor molecule 1-positive microglial activation in the hippocampus [108].

Opioid use may lead to impaired intestinal function, including weakening of the intestinal barrier and bacterial translocation, as well as alterations in the profile of microbial metabolites [2,17]. Morphine treatment promotes the translocation of *E. coli* from the intestinal lumen to the mesenteric lymph nodes and the liver, which is accompanied by increased intestinal epithelial permeability resulting from impaired intestinal barrier integrity [23]. Subcutaneous implantation of slow-release morphine pellets is widely used as an experimental model of chronic opioid exposure in rodents. It produces measurable systemic morphine levels over several days with a characteristic pharmacokinetic profile [120]. Using this experimental approach, Meng et al. [23] demonstrated that morphine up-regulates TLR2 and TLR4 expression at both mRNA and protein levels in small intestinal epithelial cells and subsequently activates TLR-dependent signaling pathways that disrupt tight junctions between enterocytes. This disruption increases intestinal barrier permeability and facilitates bacterial translocation into the host tissues. The authors demonstrated it experimentally in wild-type mice by subcutaneously implanting a 75 mg morphine pellet or a placebo pellet. Then, after 24 h, they collected mesenteric lymph node and liver suspensions, which were cultured on blood agar plates overnight for quantitation of colony-forming units (CFUs). Homogenates obtained from morphine-implanted mice contained significantly higher numbers of CFUs in contrast to animals not treated with morphine. Moreover, they proved that in MOR knockout mice, this effect did not occur. However, most available evidence derives from acute, high-dose morphine paradigms, which may not fully recapitulate the fluctuating exposure patterns characteristic of chronic human addiction. Furthermore, the pharmacokinetic profile of pellet implantation differs from that of intermittent administration, which allows greater control over dosing schedules and produces distinct patterns of drug exposure.

Alterations in the gut microbiota composition are an essential factor in the pathogenesis of many diseases, among which IBD is particularly significant. Although the etiology of IBD is complex and multifactorial, dysbiosis is associated with increased levels of *E. coli*, *Mycobacterium* spp., and *C. difficile*, which are regarded as key microorganisms linked to the development of this disease [12]. Increased intestinal permeability resulting from epithelial barrier dysfunction has been reported in clinical studies in patients with IBD, IBS, alcoholic liver disease, non-alcoholic fatty liver disease and non-alcoholic steatohepatitis, liver cirrhosis, severe acute pancreatitis, primary biliary cholangitis, type 1 and type 2 diabetes, as well as depression [19]. In line with these observations, experimental studies in animal models indicate that morphine can alter gut barrier function and host–microbial interactions, promoting bacterial translocation and infection-related outcomes, as summarized in Table 1.

## 7. Plant-Derived Psychoactive Substances as Modulators of Inflammatory and Immunological Responses Induced by *E. coli*

Although most studies indicate the immunosuppressive and anti-inflammatory properties of cannabinoids, including CBD and THC, these effects are mediated through different molecular mechanisms. They include, among others, the induction of apoptosis in selected immune cells and the limitation of activation of key mediators of the inflammatory response [123,124,125]. At the signaling level, it is suggested that the CB2 receptor plays a vital role in the modulation of the inflammatory response by cannabinoids, and that its activation in immune system cells is associated with changes in intracellular signal transduction, including, among others, regulation of MAPK-dependent pathways and effects on the expression of mediators of the inflammatory response [126]. These processes, associated with the activation of the CB2 receptor in immune system cells, may modulate the expression of genes encoding cytokines and inflammatory mediators. Reported effects include reductions in the levels of selected pro-inflammatory cytokines, such as TNF-α, interleukin-1 beta (IL-1β), IL-6, interleukin-12 (IL-12), and interleukin-23 (IL-23), enzymes involved in inflammatory responses, such as inducible nitric oxide synthase and cyclooxygenase-2, as well as specific chemokines, including C-C motif chemokine ligand 2, also known as monocyte chemoattractant protein-1, which may result in altered migration and recruitment of effector immune cells to inflamed tissues [127]. It is worth noting that pro-inflammatory cytokines induced during bacterial infections, including IL-1β, also exhibit activity within the CNS, where they are produced mainly by glial cells and play an essential role in regulating neuroimmune responses and neuromodulatory processes [20].

Experimental studies examining the effects of cannabinoids in models of inflammation induced by *E. coli* and endotoxin provide evidence that their effects on immune responses are not uniform and depend on experimental conditions. Cannabinoids have been shown to modulate inflammatory processes by regulating proinflammatory signaling pathways and producing immune mediators [124,125]. At the same time, divergent responses have been reported in some experimental models, suggesting that the biological effects of cannabinoids may vary depending on the tissue environment, the type of immune stimulus, and the experimental conditions, including differences in cannabinoid dose [124,125,128]. The context-dependent effects of CBD on immune responses indicate that the mechanisms underlying these divergent outcomes remain insufficiently understood. A summary of selected experimental studies examining these effects is presented in Table 2. Furthermore, the interpretation of cannabinoid-induced immunomodulation remains limited by several important knowledge gaps. Available evidence is largely derived from preclinical studies, while clinical data in humans remains scarce. Moreover, the pharmacological pathways of CBD, its long-term safety profile, and its interactions with other drugs are not fully characterized, and its complex pharmacokinetic properties, including low and variable bioavailability, may contribute to significant interindividual variability in response [129]. In addition, mechanistic studies across diverse immune models and stimuli are needed to understand CBD-mediated immune modulation better. The high lipophilicity of CBD and its extensive first-pass metabolism complicate dosing strategies, and the effects of varying dosages on immunomodulatory outcomes remain insufficiently defined. Long-term studies evaluating therapeutic efficacy, safety, and pharmacokinetic profiles are therefore necessary to support its clinical application [130]. These limitations complicate the interpretation of cannabinoid effects in models of inflammation induced by bacterial components such as LPS and *E. coli*.

In a retrospective cohort study based on electronic medical records, it was shown that hospitalized patients with sepsis who received opioids exhibited a significantly higher 28-day mortality compared with patients not treated with opioids. This association persisted after adjustment in multivariable analyses for age, comorbidities, clinical parameters reflecting disease severity, and pathogen type, indicating a statistically significant association between opioid exposure and mortality risk in patients with sepsis [25]. In contrast to the predominantly anti-inflammatory effects of cannabinoids, opioid exposure may be associated with impaired host defense and dysregulated endotoxin tolerance during bacterial infection (Table 3). Morphine has been shown to reduce cytokine secretion by intestinal epithelial cells in response to enteric pathogens, including *E. coli*, as well as bacterial components such as LPS and flagellin [131] Morphine may also impair phagocyte migration and activation at the mucosal surface, potentially increasing host susceptibility to enteric infections [131]. These disturbances are accompanied by impaired bacterial clearance, increased microbial translocation, and elevated endotoxin levels in the bloodstream, which may contribute to a more severe sepsis course and an increased risk of adverse clinical outcomes [132]. A study by Szkutnik-Fiedler et al. [133] demonstrated that low-dose endotoxemia induced by *E. coli*-derived LPS in a rabbit model significantly altered morphine pharmacokinetics, including faster elimination, reduced elimination half-life, and increased total body clearance. Low-grade endotoxemia was also associated with physiological changes, such as increased body temperature and reduced morphine volume of distribution, suggesting that inflammatory processes triggered by bacterial endotoxins may influence opioid pharmacokinetics.

Animal models have demonstrated that exposure to cocaine may increase susceptibility to LPS-induced bacterial endotoxemia derived from *E. coli* by enhancing sympathetic nervous system activity and modulating the inflammatory response. In a subset of individuals, this leads to disturbances in the balance of pro- and anti-inflammatory cytokines, including IL-6, IL-10, and TNF-α, and is associated with reduced survival of experimental animals [134]. These disturbances may also be relevant in clinical settings, as prenatal exposure to cocaine has been shown to promote ischemic injury to the intestines, which may facilitate secondary invasion of intestinal bacteria, including *E. coli*, and be associated with the development of systemic infection and a severe clinical course in neonates [135].

## 8. Conclusions

**Figure 3:**
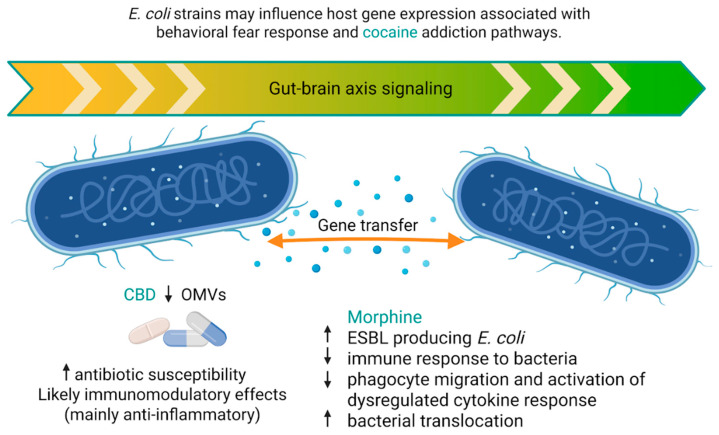
Simplified illustration of the interactions between psychoactive substances and *E. coli*. CBD, cannabidiol; OMVs, outer membrane vesicles; ESBL, extended-spectrum β-lactamases. Created in BioRender. Modrzejewska, M. (26 February 2026) https://BioRender.com/2mws1vr.

Various *E. coli* pathotypes were used in the analyzed studies, including both intestinal and extraintestinal strains. These included enterohemorrhagic *E. coli* O157:H7, a Shiga toxin-negative variant, as well as adherent-invasive *E. coli* (UM146) and other pathogenic strains differing in their ability to interact with host cells, including an intracellular strain (*E. coli* UM146) and an extracellular strain (*E. coli* UM147), as well as non-pathogenic or commensal strains used as controls. Based on the analyzed studies, no clear evidence has been demonstrated that specific classes of psychoactive substances selectively promote the growth of particular *E. coli* pathotypes. Drugs and substances such as opioids do not directly stimulate *E. coli* growth but alter host physiology, including intestinal motility, epithelial barrier integrity, and immune responses, thereby promoting bacterial systemic translocation. Figure 3 provides a schematic overview of the interactions between *E. coli* and psychoactive substances.

Available evidence indicates that naturally occurring plant derived psychoactive substances as well as opioids affect the organism not only through direct actions on the CNS but also by modulating the functioning of the microbiota–gut–brain axis. Alterations in the gut microbiota, disturbances in immune responses, and activation of inflammatory processes may constitute an essential link between exposure to psychoactive substances and long-term neurobiological consequences. In this context, *E. coli*, as a permanent component of the gut microbiota, may play a vital role, as it can, under conditions of dysbiosis and impaired immune control, enhance inflammatory signaling and influence neuroimmunological mechanisms associated with addiction development. The presented findings highlight the importance of considering interactions between psychoactive substances, the gut microbiota, and the immune system in studies on the pathogenesis of addiction and in the search for novel therapeutic strategies.

Future research should address several unresolved issues regarding opioid-induced immunomodulatory effects and their clinical relevance. Many preclinical studies investigating opioid–immune interactions rely on fixed high-dose administration protocols or sustained-release delivery systems, such as subcutaneous morphine pellets, which produce relatively stable systemic drug concentrations. However, such exposure paradigms do not fully reflect the complex patterns of opioid use observed in human addiction, characterized by fluctuating drug levels, repeated cycles of intoxication and withdrawal, and long-term behavioral and physiological adaptations. Moreover, experimental studies investigating the effects of plant-derived psychoactive substances, including cannabinoids, often employ relatively high doses in animal models that may not correspond to clinically achievable doses in humans. These limitations highlight the need for further studies evaluating how differences in pharmacokinetic profiles, exposure duration, and dosing dynamics influence immune responses, host–pathogen interactions, and inflammatory signaling pathways, particularly in the context of bacterial infection or endotoxin-induced inflammation. Consequently, findings derived from preclinical models should be interpreted with caution when extrapolating to human conditions.

## Figures and Tables

**Table 1:** Effects of morphine on bacterial translocation and sepsis-related outcomes in animal models.

Pathophysiological Mechanism	Change Related to *E. coli*	Biological Effect	Clinical Consequence/Disease Relevance	Ref.
Altered intestinal transit and bacterial overgrowth	Increased intestinal bacterial overgrowth with translocation of enteric bacteria such as *E. coli*	Passage of bacteria from the gut lumen to the mesenteric lymph nodes and systemic organs	Potential development of systemic infection and sepsis	[121]
Opioid-induced bacterial translocation	Dissemination of enteric bacteria such as *E. coli* to extraintestinal organs (liver, spleen, peritoneal cavity)	Escape of bacteria from the gastrointestinal tract and systemic spread	Potential contribution to sepsis and endotoxic shock	[122]
Immune dysregulation	Exaggerated response to LPS challenge and increased serum endotoxin levels (LPS derived from *Salmonella* subsp. I ser. *Typhimurium*)	Increased pro-inflammatory cytokine production	Accelerated progression to septic shock	[53]

**Table 2:** Effects of cannabinoids on inflammatory responses induced by *E. coli* and endotoxin.

Substance	Experimental Model	Material	Induction of Inflammation	Main Outcomes	Ref.
CBD (15 mg/kg p.o.; 30 mg/kg, p.o.; 60 mg/kg, p.o.)	Male Sprague Dawley rats	Serum and tissues (liver and kidney)	caecal slurry (400 mg/kg), LPS (100 μg/animal), and *E. coli* (0.2 mL; 2 M CFU/animal)	LPS + *E. coli* → ↑ IL-1β, IL-6, TNF-α, MMP-9; CBD ↓ inflammatory markers (strongest effect at 60 mg/kg)	[125]
CBD (5 µM), THC (5 µM)	THP-1 (ATCC TIB-202) macrophages; primary human bronchial epithelial cells	Cultured cells	LPS (0.5 µg/mL) and ATP (5 mM)	LPS → ↑ IL-6, IL-8, TNF-α; CBD and THC ↓ cytokine release	[124]
CBD (75 mg/kg, p.o.)	Female wild-type C57BL/6 mice	BALF and lung sections	Intranasal LPS (10 μg/mouse)	LPS → ↑ neutrophil infiltration and cytokine expression; CBD further ↑ pulmonary inflammation (↑ TNF-α, IL-6, IL-23)	[128]

BALF—bronchoalveolar lavage fluid; CBD—cannabidiol; IL-1β—interleukin-1 beta; IL-6—Interleukin-6; LPS—lipopolysaccharides; MMP-9—matrix metalloproteinase-9; NLRP3—NOD-like receptor family pyrin domain-containing 3; p.o.—per os (oral administration); THC—tetrahydrocannabinol; TNF-α—tumor necrosis factor alpha.

**Table 3:** Effects of opioids on host immune responses during bacterial infection.

Substance	Experimental Model	Induction of Inflammation	Main Outcomes	Ref.
Morphine (20 mg/kg, s.c.),	Balb/cJ mice (in vivo); peritoneal macrophages	LPS stimulation (1 μg/mL)	↓ TLR4 mRNA; further ↓ TLR4 expression after LPS; μ-opioid receptor–dependent effect	[106]
Chronic morphine administration	Wild-type C57BL/6 mice	LPS injection (1 mg/kg)	Biphasic IL-6 response (↓ → ↑); ↓ endotoxin tolerance → persistent inflammation; ↓ LPS-induced miR-146a (μ-opioid receptor–dependent effect)	[107]
Morphine (10 μM)	IPEC-J2 cell monolayers	*E. coli* O157:H7 (*stx*–) LPS + flagellin	↓ *E. coli*—induced IL-6 secretion; no change in pathogen-induced IL-8; ↓ LPS/flagellin-induced IL-8 secretion (opioid receptor–dependent)	[131]

IL-6—interleukin-6; IL-8—interleukin-8; LPS—lipopolysaccharides; miR-146a—microRNA-146a involved in negative regulation of Toll-like receptor signaling; s.c.—subcutaneous; *stx*–—absence of Shiga toxin genes; TLR4—toll-like receptor 4.

## Data Availability

No new data were created or analyzed in this study. Data sharing is not applicable to this article.

## Abbreviations

The following abbreviations are used in this manuscript:

2-AG	2- arachidonylglycerol
ACh	Acetylcholine
AEA	Anandamide
CB1	Cannabinoid receptor type 1
CB2	Cannabinoid receptor type 2
CBD	Cannabidiol
CFUs	Colony-forming units
CGRP	Calcitonin gene-related peptide
ClpB	Caseinolytic peptidase B
CNS	Central nervous system
CREB	cAMP response element binding protein
EECs	Enteroendocrine cells
EGCs	Enteroglial cells
ENS	Enteric nervous system
ESBL	Extended-spectrum β-lactamases
ExPEC	Extraintestinal pathogenic *E. coli*
GABA	Gamma-aminobutyric acid
GadB	Glutamate decarboxylase
IBD	Inflammatory bowel disease
IBS	Irritable bowel syndrome
IL-1β	Interleukin-1 beta
IL-6	Interleukin-6
IL-8	Interleukin-8
IL-12	Interleukin-12
IL-23	Interleukin-23
LPS	Lipopolysaccharides
MOR	µ-opioid receptor
NF-κB	Nuclear factor kappa B
OMVs	Outer membrane vesicles
QseC	Quorum-sensing *Escherichia coli* regulator C
PKA	Protein kinase A
PKC	Protein kinase C
TEER	Transepithelial electrical resistance
THC	Tetrahydrocannabinol
TLRs	Toll-like receptors
TLR4	Toll-like receptor 4
TLR5	Toll-like receptor 5
TNF-α	Tumor necrosis factor alpha
TRPV1	Transient receptor potential vanilloid
VTA	Ventral tegmental area
ZO-1, ZO-2	Zona occludens-1,2
